# An in situ tissue engineering scaffold with growth factors combining angiogenesis and osteoimmunomodulatory functions for advanced periodontal bone regeneration

**DOI:** 10.1186/s12951-021-00992-4

**Published:** 2021-08-17

**Authors:** Tian Ding, Wenyan Kang, Jianhua Li, Lu Yu, Shaohua Ge

**Affiliations:** Department of Periodontology & Biomaterials, School and Hospital of Stomatology, Cheeloo College of Medicine, Shandong University & Shandong Provincial Key Laboratory of Oral Tissue Regeneration & Shandong Engineering Laboratory for Dental Materials and Oral Tissue Regeneration, Jinan, 250012 China

**Keywords:** Periodontal bone regeneration, In situ tissue engineering, Angiogenesis, Osteoimmunomodulation, Biomimetic repair

## Abstract

**Background:**

The regeneration of periodontal bone defect remains a vital clinical challenge. To date, numerous biomaterials have been applied in this field. However, the immune response and vascularity in defect areas may be key factors that are overlooked when assessing the bone regeneration outcomes of biomaterials. Among various regenerative therapies, the up-to-date strategy of in situ tissue engineering stands out, which combined scaffold with specific growth factors that could mimic endogenous regenerative processes.

**Results:**

Herein, we fabricated a core/shell fibrous scaffold releasing basic fibroblast growth factor (bFGF) and bone morphogenetic protein-2 (BMP-2) in a sequential manner and investigated its immunomodulatory and angiogenic properties during periodontal bone defect restoration. The in situ tissue engineering scaffold (iTE-scaffold) effectively promoted the angiogenesis of periodontal ligament stem cells (PDLSCs) and induced macrophage polarization into pro-healing M2 phenotype to modulate inflammation. The immunomodulatory effect of macrophages could further promote osteogenic differentiation of PDLSCs in vitro. After being implanted into the periodontal bone defect model, the iTE-scaffold presented an anti-inflammatory response, provided adequate blood supply, and eventually facilitated satisfactory periodontal bone regeneration.

**Conclusions:**

Our results suggested that the iTE-scaffold exerted admirable effects on periodontal bone repair by modulating osteoimmune environment and angiogenic activity. This multifunctional scaffold holds considerable promise for periodontal regenerative medicine and offers guidance on designing functional biomaterials.

**Graphic Abstract:**

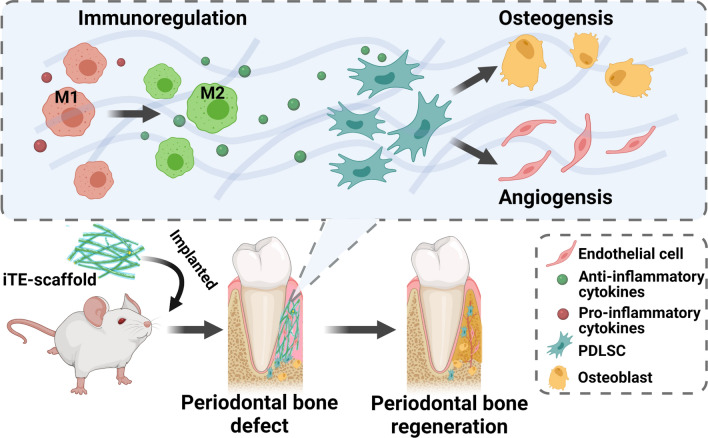

**Supplementary Information:**

The online version contains supplementary material available at 10.1186/s12951-021-00992-4.

## Background

Periodontitis is a worldwide epidemic inflammatory disease that results in the progressive destruction of periodontal tissues, including connective tissue attachment and alveolar bone [1]. And periodontal bone destruction caused by periodontitis is regarded as one of the major reasons for tooth loss, which has adverse effects on mastication and aesthetics [2]. Unfortunately, the existing therapies for periodontitis, including initial periodontal therapy, flap surgery, and guided bone regeneration, can only control disease progression while has limited capacity for bone repair [3]. The current treatments cannot completely repair the damaged periodontal bone owing to the sole focus on osteogenesis [4]. Actually, bone regeneration is a sequence of closely coordinated and overlapping processes, including inflammatory response, angiogenesis, and remodeling, which contains a lot of sophisticated cell types and signal molecules [5]. It is widely believed that immune response immediately after injury and plays a crucial part in the entire process of wound healing [6, 7]. And angiogenesis is indispensable for bone homeostasis and repair since new blood vessels not only bring nutrients but act as the pathway for inflammatory cells, mesenchymal stem cells (MSCs), and bone precursor cells to support injured bone repair [8, 9]. Thus, it is still of great scientific value to pay attention to immunomodulation and vascularization during the initial process of periodontal bone repair.

In situ tissue engineering strategy, which utilizes biomaterials to deliver multiple cytokines to take advantage of the host regenerative potential and mimic endogenous regenerative processes, has been regarded as a hopeful technology for periodontal bone defect therapy [10, 11]. Natural wound repair is a multi-step process that is orchestrated by multiple cytokines in an appropriate extracellular matrix microenvironment. This procedure can be intelligibly separate into early inflammation/angiogenesis and late tissue remodeling. Hence, appropriate combination of cytokines and release at optimal time points may be beneficial to bone tissue regeneration. Basic fibroblast growth factor (bFGF), the first discovered prototypical member of FGF family, acts as a pleiotropic part in cellular and metabolic homeostasis [12]. It is an essential growth factor (GF) in wound healing because of its benefits as a powerful inducer of vascularization [13], proliferation [14], and migration [15]. Recently, Pan et al. discovered that bFGF could reduce the production of pro-inflammatory factors and promoted lipopolysaccharide (LPS)-stimulated inflammatory macrophages polarization towards anti-inflammatory M2 phenotype through AKT/P38/NF-κB signaling pathways [16]. Another study reported that bFGF could suppress LPS-mediated inflammatory responses in periodontal ligament stem cells (PDLSCs) ex vivo [17]. Therefore, bFGF is frequently employed to rapid resolution of inflammatory responses in the tissue and promote endogenous stromal cells recruitment, proliferation, and angiogenesis for bone repair. After controlling inflammation, neovascularization, and obtaining enough cells, the later phase of tissue remodeling should be activated. Bone morphogenic protein-2 (BMP-2), one of the transforming growth factor beta (TGF-β) superfamily members, is a highly potent osteoinductive GF [18]. BMP-2 could boost the formation of Sharpey’s fibers, induce MSCs to differentiate into osteoblasts, and accelerate the regeneration and repair process of bone tissues [19]. Previously, our group has reported an in situ tissue engineering scaffold (iTE-scaffold) and found that application of bFGF followed by BMP-2 could significantly promote periodontal bone regeneration by facilitating stem cell homing, proliferation, and osteogenic differentiation [20, 21]. Because the events of immunomodulation and angiogenesis are prior to the differentiation of MSCs-osteoblast lineage during the process of bone healing, it is indispensable to explore osteoimmunomodulatory and angiogenic properties of iTE-scaffold. To the best of our knowledge, this is the first study on osteoimmunomodulatory and angiogenic effects of iTE-scaffold during periodontal bone repair.

Herein, we continue to construct a favorable iTE-scaffold for sequential delivery of bFGF and BMP-2 with the coaxial electrospinning technique. The fibrous scaffold was constructed with a poly (lactide-co-glycolide)/poly (L-lactic acid) (PLGA/PLLA) shell/core structure. PLLA was designed as the inner material due to its longer degradation rate, while PLGA was defined as the sheath owing to its adjustable and comparatively faster degradation rate. We systematically investigated the physicochemical properties and cell compatibility of the fibrous scaffolds. The osteoimmunomodulatory and angiogenic properties of iTE-scaffold were further investigated in vitro. Subsequently, the immunomodulatory function and angiogenic effect of iTE-scaffold on periodontal bone regeneration were characterized using an in vivo rat periodontal bone defect model.

## Results and discussion

### Characterization of the fibrous scaffolds

**Fig. 1:**
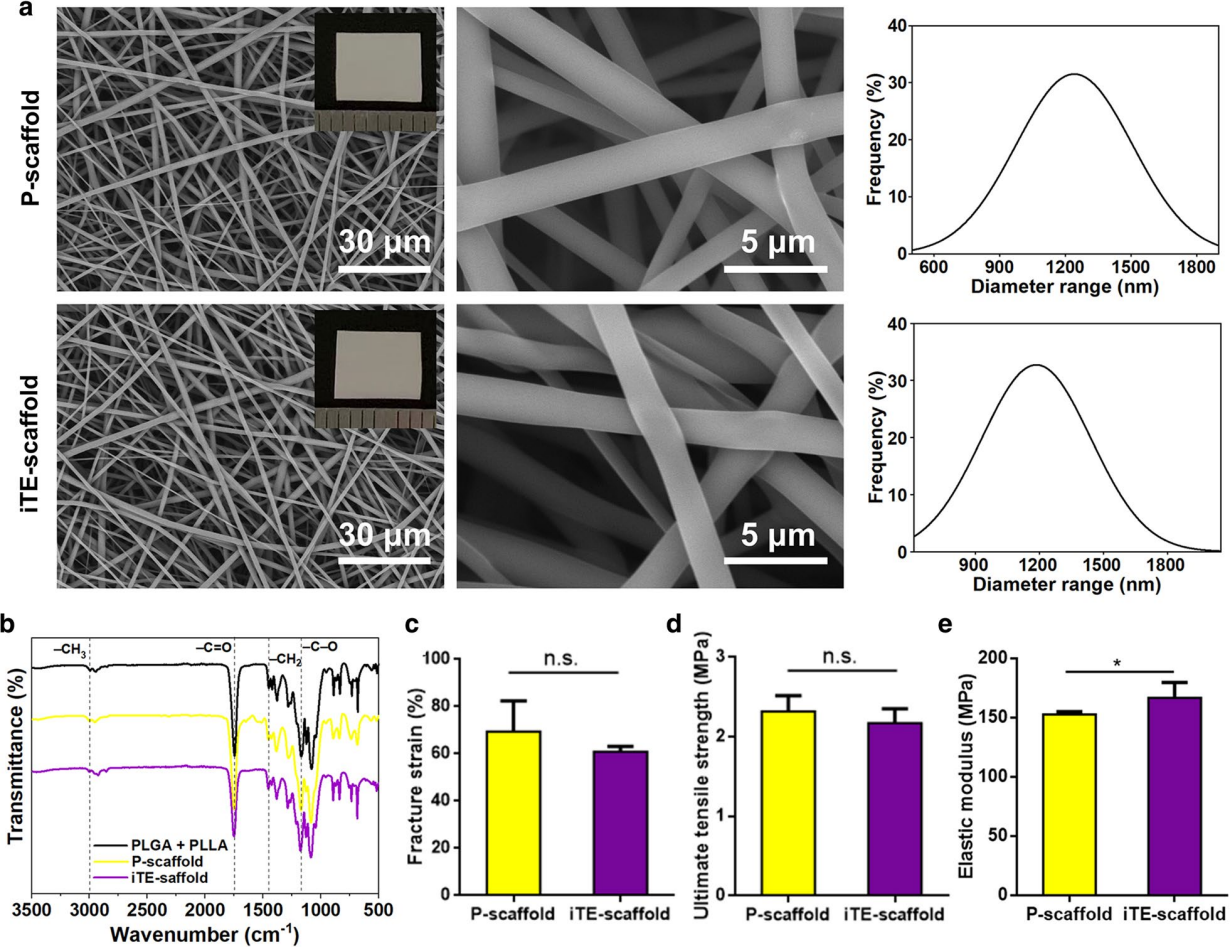
Physicochemical structure characterization assessment of the scaffolds. **a** Representative SEM images, photographs (insert), and diameter distribution analysis of P-scaffold and iTE-scaffold. **b** FTIR spectra of the scaffolds. **c** Facture strain of the fibrous scaffolds. **d** Ultimate tensile strength of the fibrous scaffolds. **e** Elastic modulus of the fibrous scaffolds. *P < 0.05,* n.s.* no statistical significance

The scaffold without GFs-loading, was defined as pristine scaffold (P-scaffold), while iTE-scaffold represented the scaffold with BMP-2 in PLLA core and bFGF in PLGA shell. To characterize the structure and surface morphology of different scaffolds, the scaffolds were visualized under scanning electron microscopy (SEM) at different magnifications. As shown in Fig. 1a, all the scaffolds were composed of randomly arranged fibers with interconnected 3D reticulated structure. Inserts of Fig. 1a were photographs of scaffolds. Although, the mean diameters of iTE-scaffold were slightly thicker than that of the P-scaffold (1198.44 nm vs 1235.27 nm). However, no statistical difference was found between their diameters (*P* > 0.05). This phenomenon revealed that the incorporation of GFs hardly changed the diameter of fibers. The fluorescence microscope was used to corroborate the presence of shell/core structure within the scaffold. As shown in Additional file 1: Fig. S1, the core and shell showed distinct red and green fluorescence, respectively, which demonstrated that all the fibrous scaffolds had typical core/sheath structures.

The surface chemical property plays a vital role in cellular regulation. Fourier transform infrared (FTIR) was conducted to evaluate the surface chemical properties and characteristic functional groups of PLLA and PLGA mixture, P-scaffold, and iTE-scaffold (Fig. 1b). As displayed by the classical spectrum of PLLA and PLGA mixture, the frequency at 2997 cm^−1^ was assigned to –CH_3_ vibration. And the peak at 1451 cm^−1^ was due to –CH_2_ bending vibration. In addition, the strong peak at 1752 cm^−1^ was corresponding to the absorption by –C=O and the frequencies at 1172 cm^−1^ belonged to –C–O stretching vibration [22]. The characteristic spectra of P-scaffold and iTE-scaffold resembled those of PLLA and PLGA mixture, and these results revealed that the electrospinning process had no significant effect on the chemical properties, excluded the influence of the diversity of material surface properties on cellular regulation [23].

It’s known to us all that the basic function of tissue engineering scaffolds is to provide adequate mechanical support for cell adhesion, growth, and tissue regeneration. Thus, the mechanical performance of the mats was evaluated by tensile testing (Fig. 1c–e). The fracture strain decreased slightly from 74.34% to 65.92% by the addition of GFs, and the ultimate tensile strength reduced a little from 3.32 MPa to 2.77 MPa. However, compared to P-scaffold group, the iTE-scaffold presented higher elastic modulus (152.76 MPa vs 166.73 MPa), and this phenomenon might be due to the incorporation of GFs diminished the polymer crystallinity. This finding suggested that loading with GFs toughened the scaffold, which was in line with former research. The possible reason was that the addition of GFs not only augmented the polymer crystallinity, but also enhanced interfacial combination between inorganic and organic phases [24]. Consequently, the iTE-scaffold can meet the standard of sufficient mechanical load capacity of tissue engineering scaffolds according to He et al.’s standard [25].

In addition, surface wettability is one of nonnegligible factors in the evaluation criteria of the biomaterials for cell adhesion and growth. Besides, Hotchkiss et al. found that macrophages cultured on hydrophilic surface exhibited more potential to differentiate towards anti-inflammatory M2 phenotype [26]. Unsurprisingly, the water contact angle (WCA) measurement results indicated that the iTE-scaffold was significantly more hydrophilic than P-scaffold, which was mostly due to the larger number of hydrophilic bioactive factors presented on the surface of fibrous mat (Additional file 1: Fig. S2). Our results were similar to a previous study, which showed that loading cytokines could ameliorate the hydrophilicity of scaffolds [18]. Consequently, as expected, GFs loading could make the iTE-scaffold more biocompatible. The release profiles of iTE-scaffold displayed a sequential release pattern, which was similar to our previous finding [20]. In brief, bFGF exhibited an initial burst release profile (over 70%) during the first few days. After that, the releasing rate of bFGF significantly slowed down and reached a standstill. On the contrary, around 4% of BMP-2 was released at the same time, because of the protection of the shell, whereas the remaining BMP-2 released continuously in the following 30 days.

### Cytocompatibility of the fibrous scaffolds

**Fig. 2:**
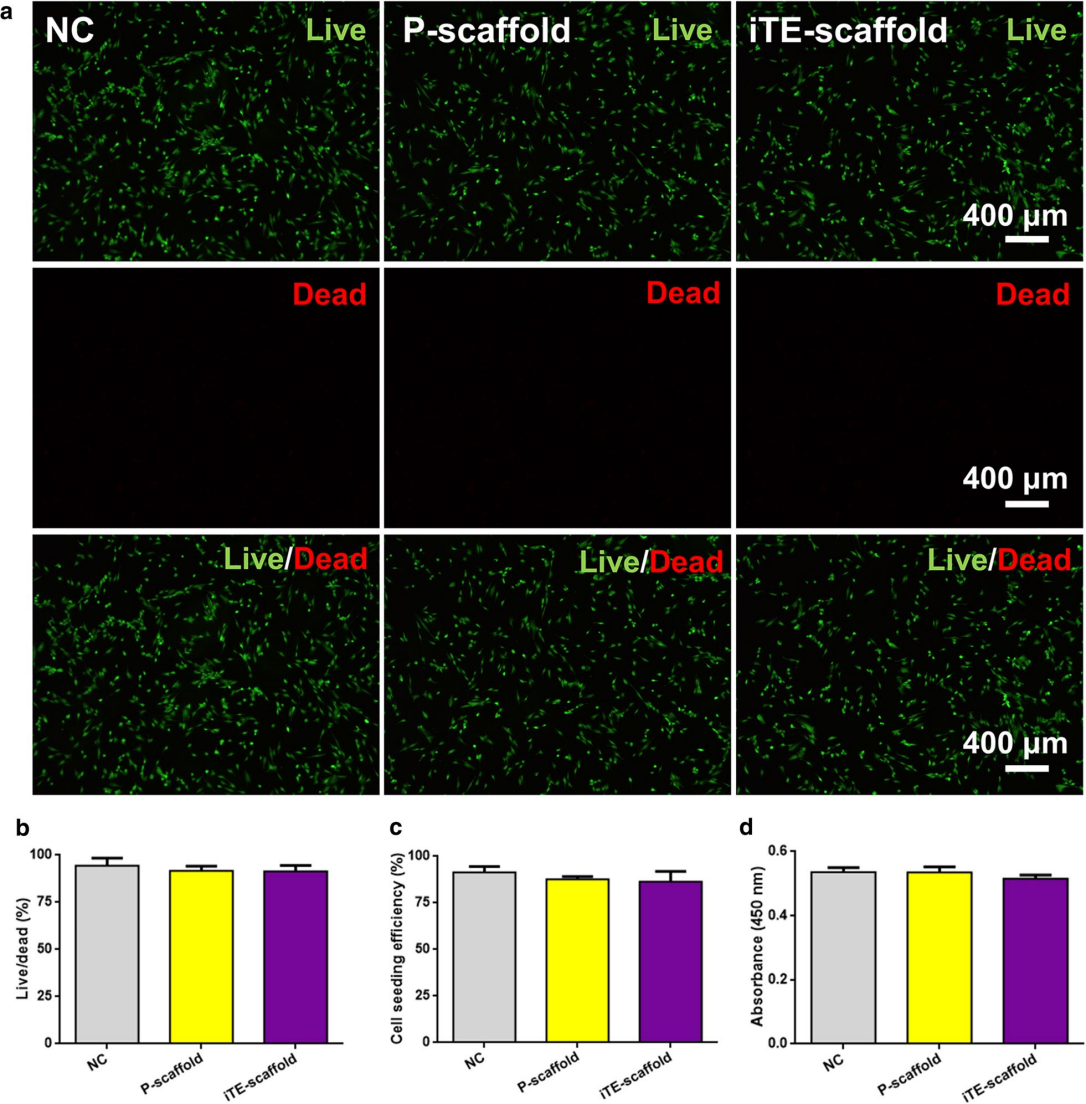
Cytocompatibility study of the scaffolds. **a**, **b** Representative images and quantitative analysis of live/dead staining of PDLSCs incubated on the different substrates. **c** Cell seeding effciency of PDLSCs cultured on the different substrates. **d** Cell activity on the different substrates

The cytotoxicity of the fibrous scaffolds was assessed by live/dead staining and cell counting kit-8 (CCK-8) assay at preset time point. As shown in Fig. 2a, b, there were almost no dead cells on P-scaffold and iTE-scaffold, suggesting the scaffolds had no adverse effect on cells. Subsequently, PDLSCs attachment on the different substrates was statistically analyzed by the determination of cell seeding density (cells/cm^2^) with respect to the density of PDLSCs initially seeded (2 × 10^4^ cells/cm^2^). Cells cultured on the tissue culture plate were served as negative control (NC). The above results indicated that cell cultured on the scaffolds exhibited high cell seeding efficiency and excellent cell adhesion similar to those on the NC (Fig. 2c). CCK-8 result was shown in Fig. 2d, and there was no statistical difference among all groups (*P* > 0.05), which further proved that the superior biocompatibility of the scaffolds. As shown in Additional file 1: Fig. S3, macrophages adhered tightly to the scaffolds with pseudopods. Interestingly, cells on the iTE-scaffold exhibited a relatively slender shape. And morphological changes in macrophages caused by topographical cues could further affect their polarization state. It has been reported that pro-healing M2 phenotype is associated with an increase in cell elongation [27]. This finding confirmed the results of surface wettability, and further verified that iTE-scaffold was in favor of tissue regeneration. Therefore, it could be seen that the fibrous scaffolds had ideal biocompatibility and could serve as an appropriate microenvironment for cell adherent and growth.

### In vitro angiogenic assessment

**Fig. 3:**
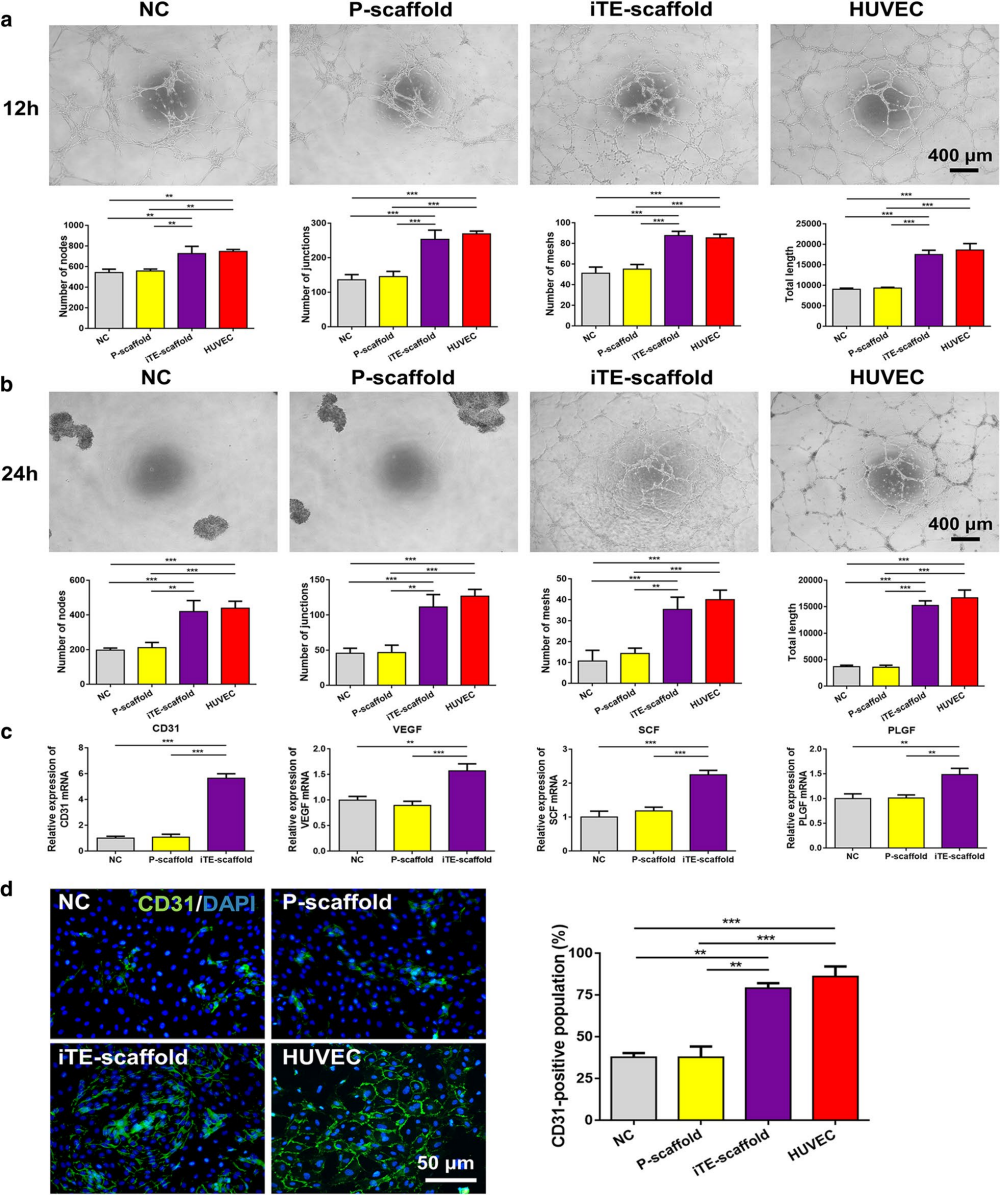
Evaluation of angiogenic capability of PDLSCs on the different fibrous scaffolds. **a**, **b** Representative images and quantitative analysis of tube-like structure formation at 12 h and 24 h after seeding on the Matrigel. Tube formation parameters: the number of nodes, junctions, meshes and total tube length. **c** Gene expression levels of *VEGF*, *CD31*, *SCF*, and *PLGF* at week 1. **d** Representative immunostaining images and quantitative analysis of CD31 in PDLSCs cultured for 7 days. **P < 0.01, ***P < 0.001

Angiogenesis is an indispensable step for bone healing, due to nutrients brought by vascular networks are essential for cells within the repair tissues, and inadequate angiogenesis can lead to cell death or undesirable tissue integration [8, 9]. The released bFGF is a strong angiogenic factor [28, 29]. Herein, the angiogenic ability of PDLSCs on different scaffolds was characterized by tube formation assay, quantitative real-time polymerase chain reaction (qRT-PCR), and immunofluorescent staining. PDLSCs cultured in endothelial cell growth medium-2 (EGM-2) were regarded as the NC, while human umbilical vein endothelial cells (HUVECs) resuspended in EGM-2 were regarded as the positive control. For tube formation assay, the iTE-scaffold group exhibited elevated angiogenic ability as showed by dramatically higher network formation parameters compared to control and P-scaffold groups. A significantly higher number of nodes, junctions, meshes, and total tube length were observed in PDLSCs cultured on iTE-scaffold at 12 h (Fig. 3a). Notably, only iTE-scaffold and HUVEC groups still remained the tubular networks, in contrast, the capillary-like networks of NC or P-scaffold groups already collapsed into spheroids after 24 h seeding on the Matrigel (Fig. 3b). In addition, the expression of angiogenic genes including *CD31*, *vascular endothelial growth factor* (*VEGF*), *stem cell factor* (*SCF*), and *placental growth factor* (*PLGF*) was further evaluated by qRT-PCR (Fig. 3c). Consistent with the results of tube formation test, the relative expression level of the angiogenic genes in PDLSCs cultured on iTE-scaffold was remarkably up-regulated compared to the other groups. Notably, compared to NC group, *CD31* mRNA expression of iTE-scaffold group was over 5 times greater. *CD31* is an important marker of vascular endothelial differentiation [30], and these results indicated the strong effect of iTE-scaffold on endothelial lineage induction. And endothelial cells played a vital role in keeping the physiological functions of blood vessels in the developed vasculatures [31]. To further validate the results, immunofluorescence staining was adopted to measure the expression level of CD31. As shown in Fig. 3d, more CD31-positive cells were presented in iTE-scaffold group, and there was no significant difference between NC and P-scaffold groups (*P* > 0.05). Collectively, all above results illuminated that iTE-scaffold maintained its angiogenic capability possibly due to the release of bFGF, and iTE-scaffold was beneficial to vascularization ex vivo, which could provide the basis for periodontal bone regeneration.

### In vitro osteoimmunomodulatory function characterization

**Fig. 4:**
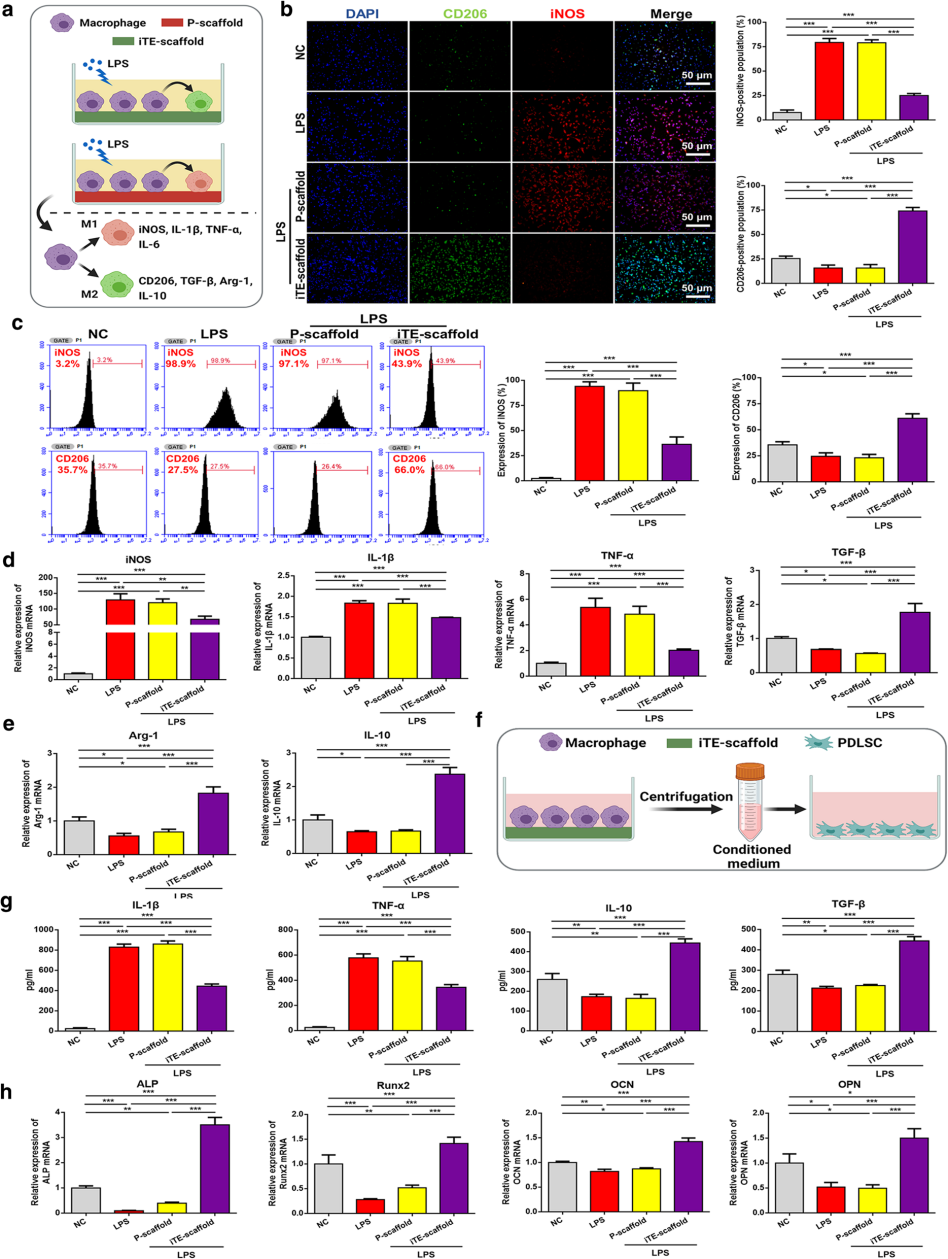
Evaluation of the osteoimmunomodulatory function in vitro. **a** Schematic illustration of biomaterial-mediated macrophage polarization. **b** Fluorescent staining images of the macrophage phenotypes and quantitative analysis of the CD206/iNOS-positive population. **c** Flow cytometric analysis of the macrophage phenotypes. **d** The gene expression level of macrophages, including *iNOS*, *TNF-α*, *IL-1β*, *TGF-β*, *Arg-1* and *IL-10*. **e** Schematic illustration of macrophage-mediated osteogenic differentiation of PDLSCs. **f** The immune factors secreted by macrophages in different groups were detected by ELISA. **g** qRT-PCR analysis for the gene expression levels of *ALP*, *Runx2*, *OCN* and *OPN* of PDLSCs cultured in the conditioned medium for 7 days. *P < 0.05, **P < 0.01, ***P < 0.001

It is commonly accepted that immune regulation is the first response that occurs after implantation and plays a vital role throughout the entire osteointegration process [7, 32]. Therefore, endowing biomaterials with admirable osteoimmunomodulatory function provides a new option to improve endogenous bone regeneration by regulating the immune homeostasis [33, 34]. Macrophages, a central regulator of immune defense during tissue healing, can be mainly divided into two phenotypes: classically M1 activated pro-inflammatory phenotype and alternatively M2 anti-inflammatory phenotype. This discrepancy is mainly due to the highly plastic property of macrophages, which could flexibly switch from one phenotype to the other in response to microenvironment signals [23]. It has been reported that the dynamic transition from M1 to M2 macrophages has a profound effect on wound repair [35, 36]. Here, we studied the effects of the iTE-scaffold on RAW264.7 macrophage polarization and the osteogenic differentiation of PDLSCs. Macrophages were treated with LPS as a simple in vitro stimulus to mimic the inflammatory microenvironment in the process of wound repair [37]. Inducible nitric oxide synthase (iNOS, an M1 phenotype marker) and CD206 (an M2 phenotype marker) were employed to characterize the macrophage phenotypes. Subsequently, we investigated the M1 and M2 phenotype distribution by immunofluorescence staining and flow cytometry. And macrophages cultivated in 10% fetal bovine serum (FBS) in Dulbecco’s modified Eagle’s medium (DMEM) without LPS denoted as the NC group, while those cultured in the inflammatory induction medium served as the LPS group. Immunofluorescence staining indicated that LPS treatment significantly increased the iNOS^+^ M1­like subpopulation, which was remarkably blunted by treatment with iTE-scaffold (Fig. 4b). Meanwhile, the proportion of CD206^+^ M2-like subpopulation was significantly up-regulated in iTE-scaffold group but unchanged in the other groups. As shown in Fig. 4c, the flow cytometry analysis result was consistent with immunofluorescence staining. In order to further explore the effect of iTE-scaffold on macrophages, qRT-PCR analysis was carried out. iTE-scaffold significantly down-regulated the expression level of M1 macrophage-related genes, including *tumor necrosis factor* (*TNF*)*-α*, *iNOS* and *interleukin* (*IL*)*-1β*, whereas up-regulated the expression of pro-healing genes that related to anti-inflammatory M2 macrophages such as *arginase-1* (*Arg-1*), *CD206* and *IL-10*, indicating that iTE-scaffold played a vital role in macrophages polarization (Fig. 4d). In addition, macrophages had a great impact on the microenvironment of bone repair sites by cytokine secretion, which both regulated inflammation and controlled cell differentiation [35]. Hence, the levels of pro­ and anti-inflammatory cytokines secreted by the treated macrophages were detected by enzyme-linked immunosorbent assay (ELISA) (Fig. 4f). Obviously, LPS remarkably promoted the release of pro-inflammatory factors (TNF-α and IL-1β), which were dramatically higher than those in the NC group. iTE-scaffold abolished the cytokine storms and distinctly stimulated the secretion of pro-healing factors (IL-10 and TGF-β), suggesting a better bone repair potential of the iTE-scaffold. It was reported that bFGF dampened the pro-inflammatory factors production and converted LPS-stimulated inflammatory macrophages polarization into M2 phenotype through AKT/P38/NF-κB pathway [16]. Furthermore, P-scaffold had no significant effect on reversing LPS-induced M1 macrophages and secretion of inflammatory factors. Collectively, these results demonstrated iTE-scaffold stimulated the transition of macrophages towards the M2 type.

To further study the osteoimmunomodulatory effect of the fibrous scaffold, the formally conditional medium of macrophages was collected to culture PDLSCs for 7 days. qRT-PCR analysis was performed to test the gene expression levels of osteogenic markers [*alkaline phosphatase* (*ALP*), *runt-related transcription factor 2* (*Runx2*), *osteocalcin* (*OCN*), and *osteopontin* (*OPN*)] to determine the indirect effect of macrophages on the osteogenic differentiation of PDLSCs. As shown in Fig. 4g, the relative mRNA level of *ALP* in the iTE-scaffold dramatically up-regulated over threefold compared to that in the NC group. *ALP* is a vital early phenotype marker during osteogenic differentiation of cells [38]. And *Runx2* expression, an important transcription factor regulating lots of other osteogenesis-related genes [39], was significantly upregulated in cells grown in condition medium collected from macrophages cultured on iTE-scaffold. Moreover, *OCN* and *OPN* expression was higher than the NC group, which might be due to *OPN* and *OCN* were mainly active during the bone matrix mineralization stage [40, 41]. These data indicated that iTE-scaffold regulated macrophages could remarkably promote the osteogenic differentiation of PDLSCs. In addition, the expression patterns of all osteogenic genes were significantly inhibited in the LPS and P-scaffold groups compared to the NC group (*P* < 0.001), suggesting that the scaffold itself cannot regulate the conversion of the macrophage phenotypes, which was in line with the above results in Fig. 4a–d. Taken together, all above findings demonstrated that M2 polarization of macrophage induced by iTE-scaffold could interact with PDLSCs and promote osteogenic differentiation.

### In vivo evaluation of angiogenesis

**Fig. 5:**
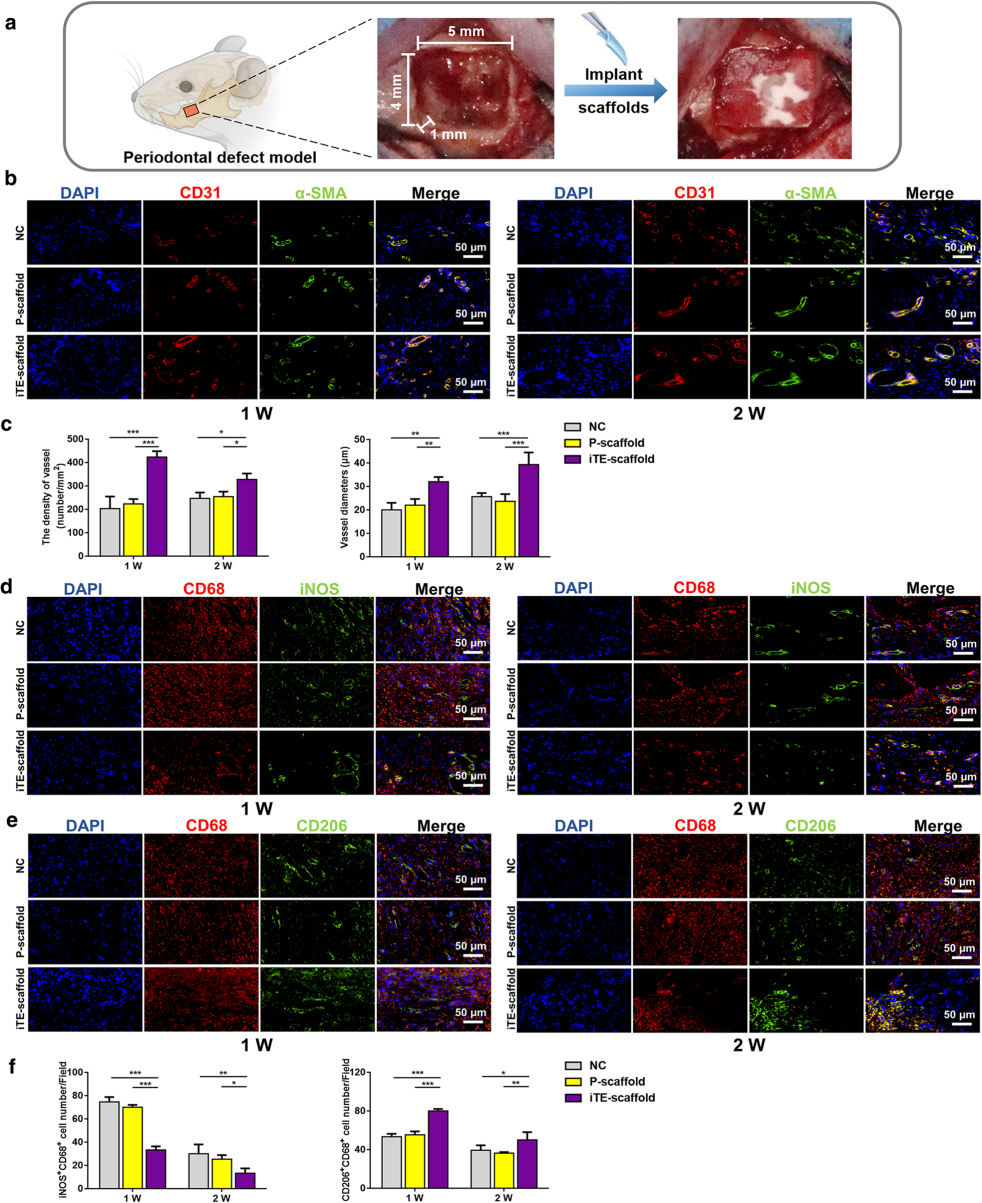
Fluorescence analysis of the tissue sections at week 1 and 2 post-operation. **a** Schematic illustration of the operation. **b** Double immunofluorescence staining of mandible sections. CD31 (red): blood vessel; α-SMA (green): smooth muscle actin. **c** Quantified neo-vascular density and neo-vascular diameters of different groups. **d**, **e** Double-labeled immunofluorescence staining images of macrophage phenotype in the defect areas. CD68 (red): a universal macrophage marker; iNOS (green): an M1-phenotype macrophage marker; CD206 (green): an M2-phenotype macrophage marker. **f** Statistical summary of the population of iNOS^+^CD68^+^ and CD206^+^CD68^+^. *P < 0.05, **P < 0.01, ***P < 0.001

The iTE-scaffold was implanted into a rat periodontal bone defect model to study its in vivo bone regenerative capacity (Fig. 5a). Bone, especially alveolar bone, is a highly vascularized tissue. Angiogenesis plays a vital role in bone homeostasis and repair because osteogenesis requires an adequate blood supply to provide oxygen and nutrition [9]. During new blood vessel formation, CD31 is strongly expressed in the endothelium of blood vessels and smooth muscle cells in the walls of blood vessels increasingly express alpha smooth muscle actin (α-SMA) [30, 42]. Therefore, to investigate the proangiogenic effect of the iTE-scaffold, sections were double-stained for CD31 (red) and α-SMA (green). As shown in Fig. 5b, c, at 1 and 2 weeks post-operation, there were lots of CD31^+^ cells gathering around α-SMA^+^ cells in the defect areas in all groups. As anticipated, significantly more blood vessels formed in iTE-scaffold group. In particular, the microvascular diameter and density were remarkably increased after iTE-scaffold implantation. These in vivo results were consistent with in vitro outcomes, indicating that iTE-scaffold possessed strong capability to promote new blood vessel formation both in vitro and in vivo and was beneficial for bone repair.

### In vivo evaluation of immunomodulatory effect

The interaction between biomaterial and immune system is a key for achieving desired bone repair effect [43]. Among numerous immune cells, macrophages exhibit a vital role in immune defense, and specific macrophage phenotype is crucial for tissue regeneration. The pro-inflammatory M1 macrophages produce IL-1β, TNF-α, iNOS, and IL-8 to mediate inflammation, and the anti-inflammatory M2 macrophages release pro-healing cytokines, like Arg-1, IL-10, TGF-β, and IL-4, to maintain tissue homeostasis and facilitate tissue repair [44]. Thus, a smooth and timely shift from pro-inflammatory M1 into pro-healing M2 macrophage is necessary for tissue repair. Accordingly, we subsequently explored the potential effect of iTE-scaffold on macrophage polarization in vivo by double-labeled fluorescence staining of CD68 (a universal macrophage marker), iNOS (an M1 marker), and CD206 (an M2 marker). As displayed in Fig. 5d–f, compared with NC or P-scaffold groups, there were more CD206^+^CD68^+^ cells and fewer iNOS^+^CD68^+^ cells in the iTE-scaffold group at 1 and 2 weeks after implantation (*P* < 0.05). It was worth noting that the number of iNOS^+^CD68^+^ cells and CD206^+^CD68^+^ cells in P-scaffold group did not show significant difference with that in NC, indicating that scaffold alone did not trigger higher pro-inflammatory response in vivo. These findings were in accordance with above in vitro study, demonstrated that iTE-scaffold could significantly promote the conversion of macrophages from pro-inflammatory M1 type to pro-healing M2 type.

Moreover, M1 and M2 phenotype type macrophages highly influenced the microenvironment of bone repair area via cytokine secretion [35]. Therefore, the expression level of the pro-inflammatory factors (TNF-α and IL-1β) and the anti-inflammatory factors (IL-10 and TGF-β) within the defective areas were detected by immunohistochemical staining. As shown in Additional file 1: Fig. S4, S5, TNF-α and IL-1β exhibited similar staining results. At week 1 and 2 post-surgery, the optical density (OD) value of TNF-α (Additional file 1: Fig. S4) and IL-1β (Additional file 1: Fig. S5) was remarkably lower in iTE-scaffold group than NC and P-scaffold. It has been reported that IL-1β and TNF-α could also inhibit the synthesis of ALP by osteoblasts and have negative effect on the secretion and mineralization of extracellular bone, which is not conducive to bone repair [45, 46]. Decreased pro-inflammatory factors might contribute to better healing outcome as high pro-inflammatory factors may lead to impaired stem cells and poor regeneration [47]. Besides, the expression levels of IL-1β and TNF-α decreased within time in each group. On the other hand, IL-10 is a pro-healing cytokine, which can inhibit the function of T helper 1 (Th1) cell and reduce the production of pro-inflammatory cytokines [48]. The expression of IL-10 gradually increased over time, and iTE-scaffold group expressed higher IL-10 than NC and P-scaffold groups (Additional file 1: Fig. S6). Moreover, TGF-β is not only the most anti-inflammatory factor, but the upstream of osteogenesis-related BMP signaling [18]. The expression level of TGF-β showed similar trends with IL-10 expression (Additional file 1: Fig. S7). In addition, there was no statistical difference in the expression of pro- and anti-inflammatory factors between P-scaffold and NC groups. This finding further indicated that fibrous scaffolds had ideal biocompatibility which was in line with our in vitro study. To sum up, all above results demonstrated that iTE-scaffold could screw macrophage towards pro-healing M2 phenotype and exert anti-inflammation effect in vivo, which was in favor of periodontal bone regeneration.

### In vivo evaluation of periodontal bone remodeling

**Fig. 6:**
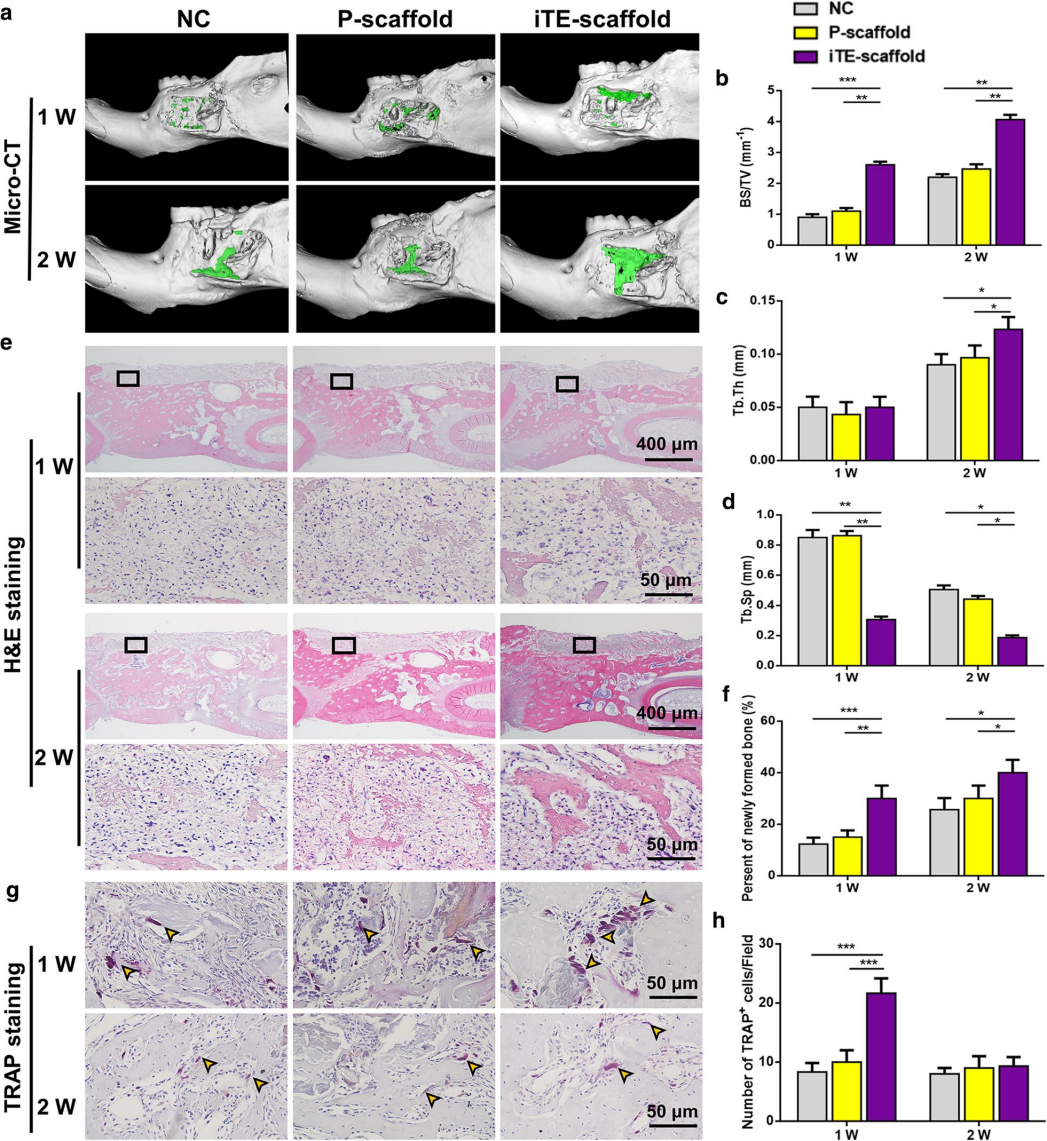
Evaluation of the bone remodeling at week 1 and 2. **a** Micro-CT reconstructed 3D images of the rat mandibular bone defects. Green color displayed the newly formed bone in the defect sites. **b**–**d** Quantification of BS/TV (**b**), Tb. Th (**c**) and Tb. Sp (**d**). **e** Representative H&E staining images of the mandible sections. The visual fields framed by the black line were magnified in the figures below. **f** The percentage of new bone formation at two time points. **g** Representative TRAP staining images of mandibular bone defects. Yellow arrows indicated osteoclasts in the trabecular bone surface. **h** The number of TRAP^+^ cells in defect regions. *P < 0.05, **P < 0.01, ***P < 0.001

The samples were analyzed with micro-computed tomography (Micro-CT) analysis and quantitative morphometric analysis after 1 and 2 weeks post-operation. The representative images of 3D digital reconstruction showed that iTE-scaffold presented the largest osteogenic area at all time points (Fig. 6a). Subsequently, we quantitatively and qualitatively characterized the new bone formation in defect areas based on reconstructed 3D Micro-CT images. Significantly, more bone surface (BS) was observed in iTE-scaffold group (Fig. 6b), which was coincident with Micro-CT images. Additionally, the iTE-scaffold group exhibited a greater trabecular bone thickness (Tb. Th) than NC and P-scaffold groups at week 2 (Fig. 6c), and simultaneously, as shown in Fig. 6d, the value of trabecular bone separation (Tb. Sp) reduced over time compared to the other groups. The above finding revealed that more mature and denser bone formed in the iTE-scaffold group.

To further study the periodontal bone regeneration capability of the scaffolds, histological analysis of new bone formation was performed through hematoxylin and eosin (H&E) staining (Fig. 6e, f). In line with the Micro-CT analysis results, a number of new bone formation could be observed in the bone defect areas of iTE-scaffold group, while those in the other groups were barely observed at 1 week after implantation. There were more newly formed bones in the defect areas in all groups at week 2. All the above results proved that iTE-scaffold exhibited remarkable bone repair performance for periodontal bone defect.

In addition, bone remodeling depends on the homeostatic balance between bone formation (osteoblasts) and bone resorption (osteoclasts) [49, 50]. And the initiation and functionalization of osteoclasts play a necessary role in bone remodeling and repair. Thus, the osteoclast activity within the defect areas was evaluated by tartrate-resistant acid phosphatase (TRAP) staining. As shown in Fig. 6g, h, the number of TRAP^+^ cells in iTE-scaffold group was over two folds as much as that in NC group at week 1 post-surgery (*P* < 0.001). However, the number of TRAP^+^ cells decreased markedly at week 2 and no significant difference was found among all groups (*P* > 0.05). These results revealed that iTE-scaffold initiated early osteoclastogenesis. Generally, bone apposition occurs at sites where bone resorption has recently occurred, because these sites were prone to attract precursor bone cells and express rich bone matrix proteins for later osteogenic differentiation [50]. Therefore, osteoclastogenesis, even if transient, may be necessary for iTE-scaffold induced anabolism during bone reconstruction.

## Conclusions

In conclusion, we fabricated a novel scaffold based on in situ tissue engineering strategy. The iTE-scaffold was proved for the first time to effectively modulate immune response and angiogenic activity in vitro and in vivo. The iTE-scaffold could significantly steer macrophage polarization towards the pro-healing M2 phenotype and enhance vascularization. Moreover, this immunomodulatory effect could facilitate the osteogenic differentiation of PDLSCs. When the fibrous scaffolds were implanted in the periodontal bone defect model, the iTE-scaffold exhibited an anti-inflammatory response, provided adequate blood supply, and obtained desired bone repair outcome. Collectively, this novel functional iTE-scaffold opens new doors for in situ tissue engineering.

## Materials and methods

### Preparation of the scaffolds

Poly (lactide-co-glycolide) (LA:GA = 50:50) and poly (L-lactic acid) were supplied by Daigang Biomaterial Co., Ltd, Jinan, China. BMP-2 and bFGF were sourced from PeproTech (Rocky Hill, USA). 0.15 g PLLA and 1 g PLGA were added to 1.25 mL and 5 mL 1,1,1,3,3,3-hexafluoro-2-propanol (HFIP, Macklin, Shanghai, China) so as to make the PLLA core solution and PLGA sheath solution, respectively. After stirring overnight, BMP-2 in phosphate buffered saline (PBS, 0.01 M, HyClone, Logan, USA) (1 μg, 0.125 mL) was added into the core solution by stirring and sonicating, while bFGF in PBS (500 ng, 0.5 mL) was slowly added into the sheath solution in the same way according to our previous study [20]. In addition, the control group selected PBS as the water phase, which was prepared under similar parameters. Then, core and sheath solutions were added into two independent syringes which were connected by a coaxial metal needle. The flow rates of the inner and outer fluids were 0.5 mL/h and 2 mL/h, respectively. And the 15 kV electric potential was used in this study. The electrospinning process was conducted under ambient conditions (25 ± 2 °C with relative humidity 50 ± 5%). Two kinds of scaffolds were fabricated. The scaffold was about 1 mm in thickness, dominated by the electrospinning time. The control group, representing simple PBS without GFs, was defined as pristine scaffold (P-scaffold) group. And iTE-scaffold group denoted the scaffold with bFGF (sheath) and BMP-2 (core). Finally, the collected membranes were preserved at − 20 °C for future experiments.

### Characterizations of the scaffolds

The surface morphology and structure of the scaffolds was acquired under SEM (S-4800, Hitachi, Tokyo, Japan). To corroborate the shell/core structure of fibrous scaffold, the fluorescence dyes Rhodamine B (red) and Coumarin 6 (green) (Sigma-Aldrich, USA) were subsequently added into core and shell solutions, respectively. Afterwards, the distribution of fluorescence dyes in collected fibers was observed by a fluorescence microscope (Leica, Wetzlar, German). The chemical composition of PLLA and PLGA mixture, P-scaffold and iTE-scaffold was evaluated by the FTIR spectroscopy (Thermo Fisher Scientific, Waltham, USA). The mechanical properties of scaffolds were characterized utilizing universal testing machine (Instron, Boston, USA). The surface wettability of the scaffolds was assessed using a pendant drop method on the WCA analyzer (DSA10, Kruss, Hamburg, Germany).

### Cell culture

The isolation of human PDLSCs was carried out based on our previously procedures [51]. Primary PDLSCs were maintained in DMEM (HyClone) containing 20% FBS (BioInd, Kibbutz, Israel). PDLSCs were passaged using trypsin–EDTA solution (HyClone) until 90% confluent cells were reached. Afterwards, the passaged cells were cultivated with 10% FBS in DMEM.

RAW264.7 macrophages were sourced from the Shanghai Cell Bank of the Chinese Academy of Sciences and cultivated in 10% FBS-supplemented DMEM. HUVECs were commercially purchased (ScienCell, San Diego, USA) and seeded in supplemented endothelial culture medium (ECM, ScienCell). All cells were cultured at a 37 °C and 5% CO_2_ incubator with a humidified atmosphere.

### Cell viability evaluation

The compatibility of PDLSCs seeded on tissue culture plate, P-scaffold and iTE-scaffold was evaluated by a LIVE/DEAD viability/cytotoxicity kit (Invitrogen, CA, USA). Cells cultured on the tissue culture plate were served as NC. After incubation for 24 h, propidium iodide (PI) and calcein acetoxymethyl ester (AM) were used to stain the dead and live cells, respectively. Afterwards, the samples were observed under the fluorescence microscope. Meanwhile, cell adhesion assay of the fibrous scaffolds was performed as previously reported [52]. Cell viability and proliferation on different substrates were evaluated by CCK-8 (Dojindo Laboratories, Tokyo, Japan) after cultivated for 24 h. In addition, RAW264.7 macrophages were seeded on the scaffolds. And the morphology of macrophages on P-scaffold and iTE-scaffold was characterized with an SEM after dehydration by gradient alcohol.

### In vitro vascularization assay

PDLSCs were cultured with EGM-2 (Lonza, Walkersville, USA) on different membranes for 7 days. In vitro angiogenic capacity was assessed by the tube formation assay on Matrigel (354230, Corning, NY, USA) as before [48]. PDLSCs in EGM-2, PDLSCs on P-scaffold, PDLSCs on iTE-scaffold and HUVECs were collected and resuspended in the medium of EGM-2. Then, cell suspensions (5 × 10^4^ cells) in 100 μl EGM-2 were reseeded onto Matrigel-coated wells. PDLSCs cultured in EGM-2 regarded as the NC, while HUVECs resuspended in EGM-2 regarded as the positive control. Tube formation was observed at predetermined time points (12 h, 24 h) under the inverted light microscope (Olympus, Tokyo, Japan). Subsequently, the following angiogenic parameters, including total length, the number of junctions, meshes and nodes were evaluated by Image J software (NIH, USA).

The relative gene expression levels of angiogenic differentiation (*CD31*, *VEGF*, *PLGF* and *SCF*) were analyzed by qRT-PCR. And *Glyceraldehyde‐3‐phosphate dehydrogenase* (*GAPDH*) primer was selected as the housekeeping gene and primer sequences were provided in Additional file 1: Table S1.

In addition, for fluorescence analysis, the samples were fixed with paraformaldehyde (4%) and blocked with normal goat serum for 40 min. Thereafter, samples were incubated with rabbit anti-CD31 (1:200, ab32457, Abcam, Cambridge, UK) overnight. The samples were further incubated with CoraLite488-conjugated goat anti-rabbit secondary antibody (1:800, SA00013-2, Proteintech, Chicago, IL, USA) and then counterstained with DAPI (ab104139, Abcam). The images were captured with the fluorescence microscope (Olympus).

### In vitro osteoimmunomodulatory function characterization

#### Inflammatory response of macrophages

RAW264.7 macrophages were collected, counted, and resuspended in the inflammatory induction medium [10% FBS DMEM supplemented with 100 ng/mL LPS (Sigma-Aldrich)] according to a previous study [37]. Subsequently, cell suspensions (2 × 10^5^ cells) were seeded onto the P-scaffold and iTE-scaffold within each well in 6-well plates. Moreover, RAW264.7 cultivated in 10% FBS in DMEM without LPS denoted as the NC group, while those cultured in the inflammatory induction medium served as the LPS group. The induction medium was collected after cultivation for 24 h and replaced with 6 mL DMEM. After cultivation for 6 h, the corresponding medium was collected as the conditioned medium for further experiments. Subsequently, the macrophages were double-stained with primary antibodies of anti­mannose receptor antibody (1:300, ab64693, Abcam) and iNOS antibody (1:200, ab49999, Abcam). The fluorescence images were obtained under the fluorescence microscope. Meanwhile, the expression of M1/M2 macrophage surface markers was assessed by flow cytometry (Accuri-C6, BD Biosciences, San Diego, USA). Stimulated macrophages were incubated with allophycocyanin (APC)*-*conjugated anti-iNOS (2102823, Invitrogen) and anti-CD206 (MMR) (141707, Biolegend). Related conjugated IgG was used as control. Subsequently, the gene level was measured by qRT*-*PCR. The detected genes included *TNF-α*, *iNOS*, *Arg-1*, *CD206, IL-10*, *IL-1β* and *TGF-β*. And the primer sequences were showed in Additional file 1: Table S2. Besides, the concentration of anti- and pro- inflammatory cytokines (IL-10, TGF-β, IL-1β and TNF-α) in collected induction medium were detected with corresponding ELISA kits (Biolegend) based on the manufacturer’s guidance.

#### Osteogenic differentiation of PDLSCs

To assess whether macrophages in response to membranes could regulate osteogenic differentiation of PDLSCs. Cells (2 × 10^5^ cells per well) were seeded into the 6-well plate and stimulated with conditioned medium to mimic the immune environment in vivo. After cultured for 7 days, the osteogenesis-related genes in PDLSCs were evaluated by qRT-PCR. The sequences of the primers of *ALP*, *Runx2*, *OCN* and *OPN* were showed in the Additional file 1: Table S3.

### Establishment of mandibular periodontal bone defect model

A total of eighteen eight-week-old male Wistar rats were used in this study. The rats were randomly divided into NC, P-scaffold, and iTE-scaffold groups. Surgical procedures were conducted as previously described [53]. In brief, after shaving and cleaning, the buccal surface of the mandible was exposed. Then, a 5 × 4 × 1 mm defect was created by a fissure bur. The defect area located about 1 mm behind the front border of the mandible and 1 mm below the upper border of the mandible. Subsequently, the defects were filled with iTE-scaffold and P-scaffold based on the respective groups, whereas the defect areas left untreated defined as the NC group. The rats were euthanized at week 1 and 2 post-surgery, the samples were harvested and fixed for the following analysis.

### Micro-CT analysis

To evaluate the bone regeneration in the defect area, the harvested mandibles were scanned by high-resolution Micro-CT (PerkinElmer, MA, USA). Samples were reconstructed and analyzed using supporting software. The various bone parameters, including BS/TV (%), Tb. Sp (mm) and Tb. Th (mm) were analyzed.

### Histology, immunohistochemistry (IHC) and immunofluorescence analyses

The samples were decalcified in 10% disodium ethylenediaminetetraacetic acid (EDTA-Na_2_, Solarbio, Beijing, China) at 4 °C for 30 days. Afterwards, decalcified samples were longitudinally embedded in paraffin wax and sliced into 5 μm sections. Then, the tissue sections were stained with H&E staining kit (Solarbio) and TRAP staining kit (Solarbio) based on the manufacturer’s instructions. The stained sections were photographed under the microscope (BX53, Olympus) and analyzed with Image Pro­Plus 6.0 Software (Media Cybernetics, Silver Spring, USA).

IHC staining was performed with an anti-Rabbit HRP/DAB Detection Kit (ab64261, Abcam) following the manufacturer’s protocol. The primary antibodies used anti-TNF-α (1:200, AF7014, Affinity, OH, USA), anti-IL-1β (1:200, AF5103, Affinity), anti-IL-10 (1:300, DF6894, Affinity), TGF-β (1:200, AF1027, Affinity). The images were photographed under the microscope. For semiquantitative analysis, mean OD of IL-10, TNF-α, IL-1β and TGF-β was measured by Image Pro-Plus 6.0 software.

Immunofluorescent staining was performed as described before [54]. In order to detect phenotype switching of macrophages and angiogenesis in vivo, the tissue sections were incubated with primary antibodies of anti-CD68 (1:250, ab955, Abcam)/anti-iNOS (1:200, ab15323, Abcam), anti-CD68/anti-CD206 and anti-α-SMA (1:400, ab7817, Abcam)/anti-CD31 (1:300, ab28364, Abcam). The fluorescent slides were finally covered by the cover glass using mounting medium containing DAPI. The fluorescent images were obtained with the fluorescence microscope, and the number of CD206^+^CD68^+^ cells, iNOS^+^CD68^+^ cells, α-SMA^+^ cells and CD31^+^ cells in the defects were counted and measured by Image J software.

### Statistical analysis

All statistical data were shown as mean ± standard deviation (SD). Tests were analyzed using GraphPad Prism software (Version 6, Boston, USA). Differences between two groups were performed by Student’s t-test, and multiple group comparisons were performed by one-way or two-way ANOVA. And Turkey HSD comparison test was utilized when multiple comparisons were performed. Differences were considered to be significant at *P* < 0.05.

## Supplementary Information

**Additional file 1: Table S1.** Primer sequences for qRT-PCR. **Table S2.** Primer sequences for qRT-PCR. **Table S3.** Primer sequences for qRT-PCR. **Fig. S1.** Fluorescence microscopy images of the fibrous scaffolds after fluorescent staining. **Fig. S2.** WCA of different fibrous membranes. **Fig. S3.** SEM images of macrophages cultured on P-scaffold and iTE-scaffold for 24 h. **Fig. S4.** Immunohistochemistry analysis of TNF-α expression in all groups. **Fig. S5. **Immunohistochemistry analysis of IL-1β expression in all groups. **Fig. S6.** Immunohistochemistry analysis of IL-10 expression in all groups. **Fig. S7.** Immunohistochemistry analysis of TGF-β expression in all groups.

## Data Availability

The datasets in the current study are included in the published article or available from the corresponding author on reasonable request.

## Abbreviations

iTE-scaffold: In situ tissue engineering scaffold
P-scaffold: Pristine scaffold
PLGA: Poly (lactide-co-glycolide)
PLLA: Poly (L-lactic acid)
bFGF: Basic fibroblast growth factor
BMP-2: Bone morphogenetic protein-2
PDLSC: Periodontal ligament stem cell
HUVEC: Human umbilical vein endothelial cell
LPS: Lipopolysaccharide
SEM: Scanning electron microscopy
FTIR: Fourier transform infrared spectroscopy
WCA: Water contact angle
DMEM: Dulbecco’s modified Eagle’s medium
ECM: Endothelial culture medium
VEGF: Vascular endothelial growth factor
PLGF: Placental growth factor
SCF: Stem cell factor
GAPDH: Glyceraldehyde‐3‐phosphate dehydrogenase
qRT-PCR: Quantitative real-time polymerase chain reaction
iNOS: Inducible nitric oxide synthase
TGF-β: Transforming growth factor beta
TNF-α: Tumor necrosis factor-α
Arg-1: Arginase-1
IL-10/1β: Interleukin-10/1 beta
ELISA: Enzyme-linked immunosorbent assay
ALP: Alkaline phosphatase
Runx2: Runt-related transcription factor 2
OCN: Osteocalcin
OPN: Osteopontin
Micro-CT: Micro-computed tomography
BS/TV: Bone surface area/total volume
Tb. Sp: Trabecular bone separation
Tb. Th: Trabecular bone thickness
H&E: Hematoxylin and eosin
TRAP: Tartrate-resistant acid phosphatase
IHC: Immunohistochemistry
Fig.: Figure
