# Supplementary material for: An in situ tissue engineering scaffold with growth factors combining angiogenesis and osteoimmunomodulatory functions for advanced periodontal bone regeneration
Source: J Nanobiotechnology. 2021 Aug 17;19:247. doi: 10.1186/s12951-021-00992-4 (PMC8371786; doi:10.1186/s12951-021-00992-4)
Supplement: Supplementary file 1 — Additional file 1: Table S1. Primer sequences for qRT-PCR. Table S2. Primer sequences for qRT-PCR. Table S3. Primer sequences for qRT-PCR. Fig. S1. Fluorescence microscopy images of the fibrous scaffolds after fluorescent staining. Fig. S2. WCA of different fibrous membranes. Fig. S3. SEM images of macrophages cultured on P-scaffold and iTE-scaffold for 24 h. Fig. S4. Immunohistochemistry analysis of TNF-α expression in all groups. Fig. S5. Immunohistochemistry analysis of IL-1β expression in all groups. Fig. S6. Immunohistochemistry analysis of IL-10 expression in all groups. Fig. S7. Immunohistochemistry analysis of TGF-β expression in all groups. [file 12951_2021_992_MOESM1_ESM.docx]

***Supporting Information***

**An *In Situ* Tissue Engineering Scaffold with Growth Factors Combining Angiogenesis and Osteoimmunomodulatory Functions for Advanced Periodontal Bone Regeneration**

**Tian Ding,** **Wenyan Kang, Jianhua Li, Lu Yu, Shaohua Ge****^*^**

*Department of Periodontology & Biomaterials, School and Hospital of Stomatology, Cheeloo College of Medicine, Shandong University & Shandong Provincial Key Laboratory of Oral Tissue Regeneration & Shandong Engineering Laboratory for Dental Materials and Oral Tissue Regeneration, Jinan 250012, China*

^*^ **Corresponding authors**: *E-mail address*: shaohuage@sdu.edu.cn (S. Ge)

**Table S1.** **Primer sequences for qRT-PCR.**

| **Genes** | **Primers sequences** |
| --- | --- |
| *TNF-α* | 5’- CAGGCGGTGCCTATGTCTC -3’ |
|  | 5’- CGATCACCCCGAAGTTCAGTAG -3’ |
| *IL-1β* | 5’- TGCCACCTTTTGACAGTGATG -3’ |
|  | 5’- TGATGTGCTGCTGCGAGATT -3’ |
| *iNOS* | 5’- CTCACTGTGGCTGTGGTCACCTA -3’ |
|  | 5’- GGGTCTTCGGGCTTCAGGTTA -3’ |
| *IL-10* | 5′-GCCAGAGCCACATGCTCCTA-3′ |
|  | 5′-GATAAGGCTTGGCAACCCAAGTAA-3′ |
| *Arg-I* | 5’- TGTCCCTAATGACAGCTCCTT -3’ |
|  | 5’- GCATCCACCCAAATGACACAT -3’ |
| *TGF-β* | 5’- CTAAGGCTCGCCAGTCCCC -3’ |
|  | 5’- TGCGTTGTTGCGGTCCAC -3’ |
| *GADPH* | 5’- TGACCACAGTCCATGCCATC -3’ |
|  | 5’- GACGGACACATTGGGGGTAG -3’ |

**Table S2.** **Primer sequences for qRT-PCR.**

| **Genes** | **Primers sequences** |
| --- | --- |
| *ALP* | 5’- ATGGGATGGGTGTCTCCACA -3’ |
|  | 5’- CCACGAAGGGGAACTTGTC -3’ |
| *Runx2* | 5’- TCCACACCATTAGGGACCATC -3’ |
|  | 5’- TGCTAATGCTTCGTGTTTCCA -3’ |
| *OCN* | 5’- TCACACTCCTCGCCCTATT -3’ |
|  | 5’- GATGTGGTCAGCCAACTCG -3’ |
| *OPN* | 5′- TCCTAGCCCCACAGACCCTT -3′ |
|  | 5′- CACACTATCACCTCGGCCAT -3′ |
| *GADPH* | 5’- TGACCACAGTCCATGCCATC -3’ |
|  | 5’- GACGGACACATTGGGGGTAG -3’ |

**Table S3.** **Primer sequences for qRT-PCR.**

| **Genes** | **Primers sequences** |
| --- | --- |
| *CD31* | 5’- AAGCTGCCGGTTCTTAAATCC -3’ |
|  | 5’- AACTTGGTGGAAGGAGGGTATG -3’ |
| *VEGF* | 5’- CACAGTGGCCGACACCTAAA -3’ |
|  | 5’- TCTGACCATGTTGGCCAGACT -3’ |
| *SCF* | 5’- GACCTTGTGGAGTGCGTGAA -3’ |
|  | 5’- CTGGGTTCTGGGCTCTTGAAT -3’ |
| *PLGF* | 5′- TTGTCTGCTGGGAACGGCTCGT -3′ |
|  | 5′- CCGGCACACAGTGCAGATTCT -3′ |
| *GADPH* | 5’- TGACCACAGTCCATGCCATC -3’ |
|  | 5’- GACGGACACATTGGGGGTAG -3’ |


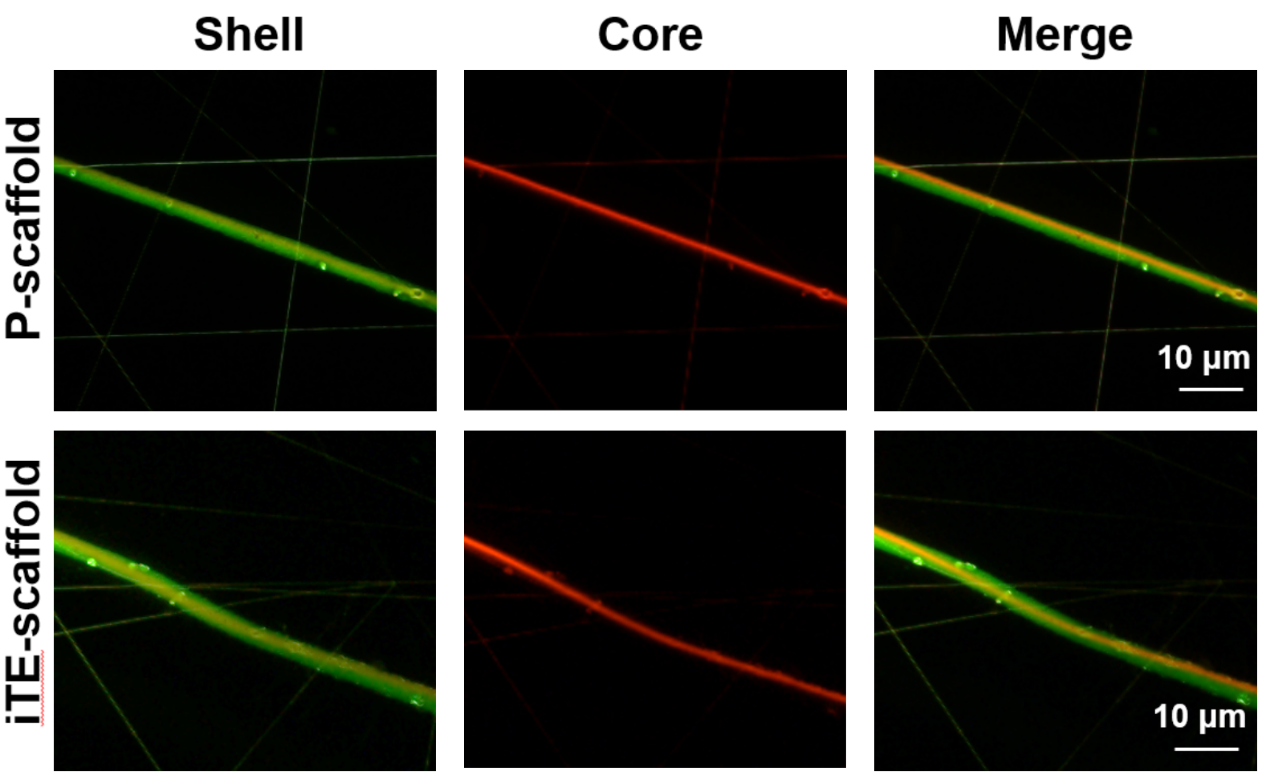


**Fig. S1. Fluorescence microscopy images of the fibrous scaffolds after fluorescent staining.** The PLLA core is indicated in red, while PLGA shell in green. Scale bar: 10 μm.


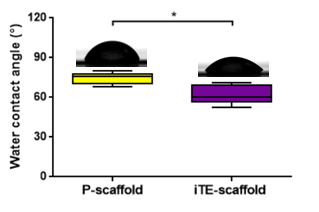


**Fig. S2.** **Water contact angle of different fibrous membranes.** ^*^*P* < 0.05


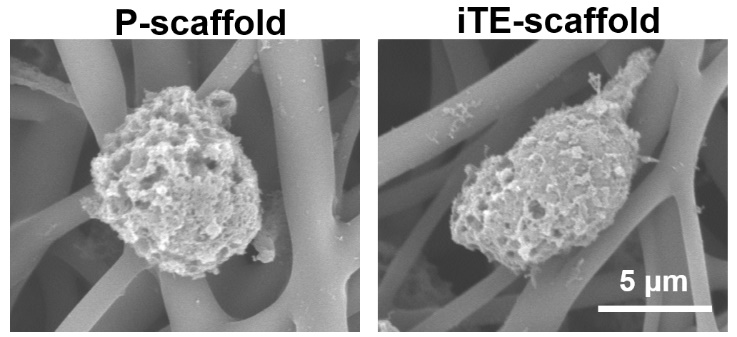


**Fig. S3. SEM images of macrophages cultured on P-scaffold and iTE-scaffold for 24 h.**


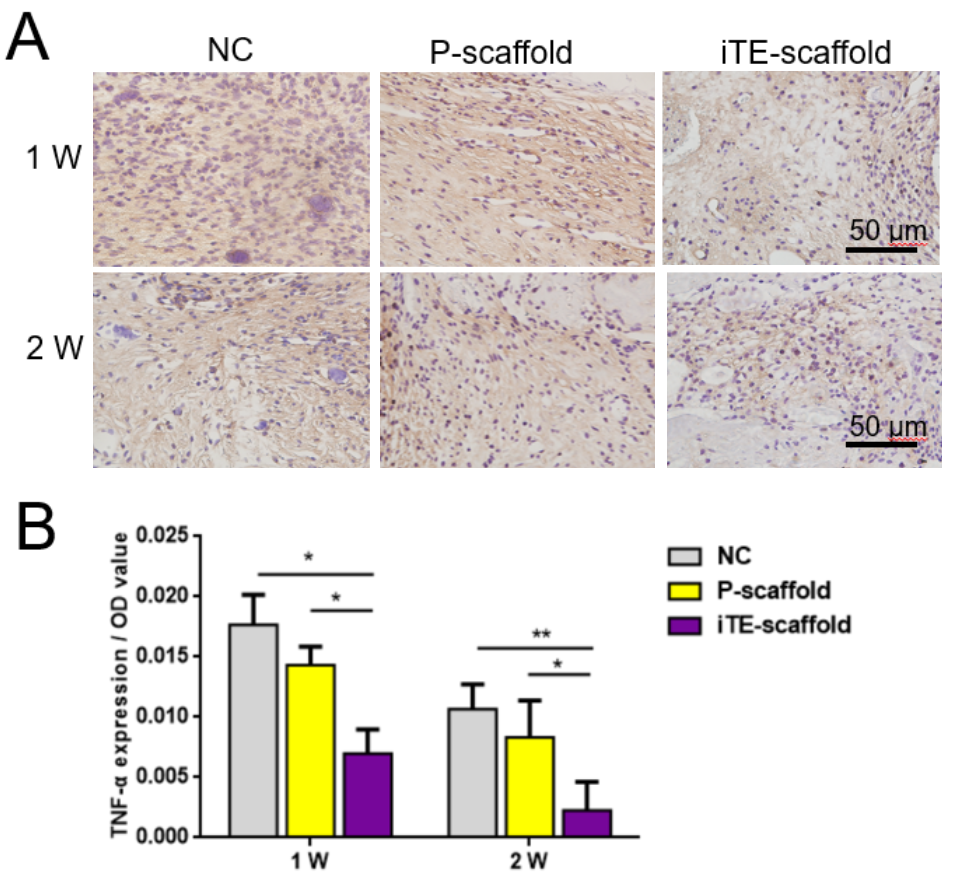


**Fig. S4. Immunohistochemistry analysis of TNF-α expression in all groups.** (A) Immunohistochemical staining of TNF-α(brown) at week 1 and 2 post-operation. Scale bar: 50 μm. (B) Quantitative analysis of TNF-αexpression at week 1 and 2 post-operation. ^*^*P* < 0.05 and ^**^*P* < 0.01.


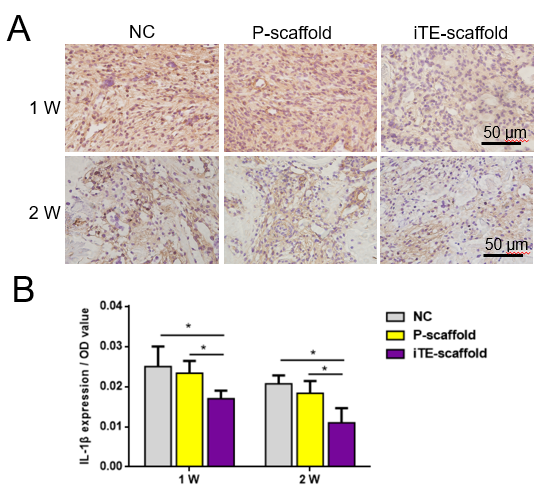


**Fig. S5. Immunohistochemistry analysis of IL-1β expression in all groups.** (A) Immunohistochemical staining of IL-1β (brown) at week 1 and 2 post-operation. Scale bar: 50 μm. (B) Quantitative analysis of IL-1β expression at week 1 and 2 post-operation. ^*^*P* < 0.05.


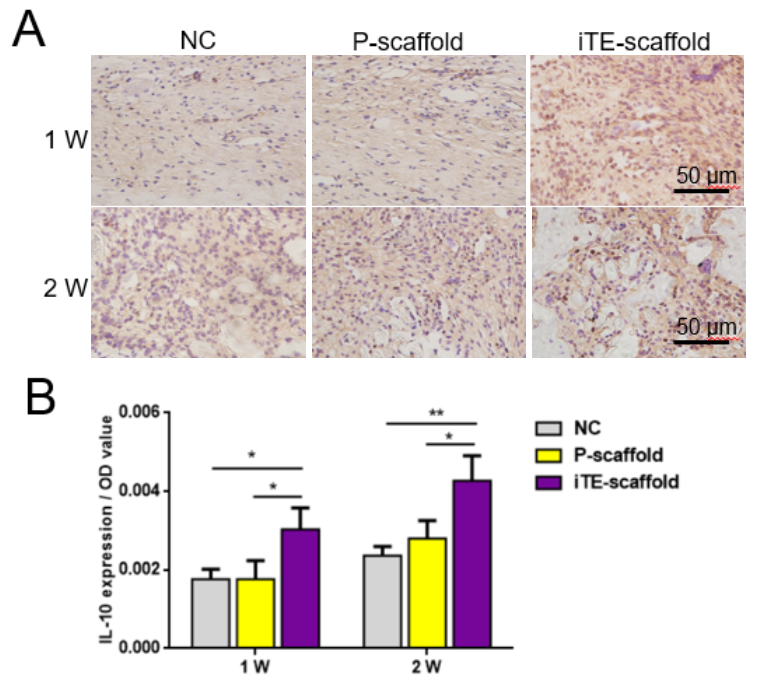


**Fig. S6. Immunohistochemistry analysis of IL-10 expression in all groups.** (A) Immunohistochemical staining of IL-10 (brown) at week 1 and 2 post-operation. Scale bar: 50 μm. (B) Quantitative analysis of IL-10 expression at week 1 and 2 post-operation. ^*^*P* < 0.05 and ^**^*P* < 0.01.


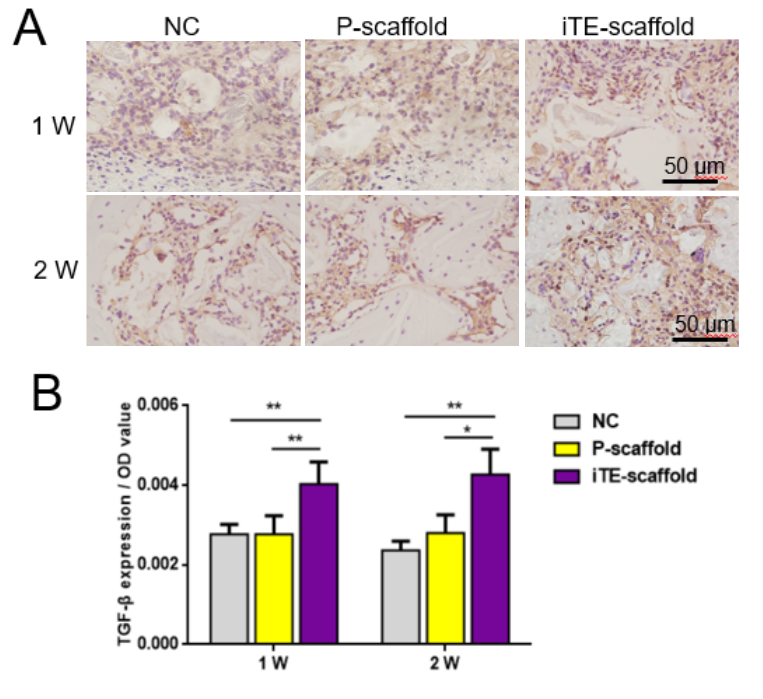
 **Fig. S7. Immunohistochemistry analysis of TGF-β expression in all groups.** (A) Immunohistochemical staining of TGF-β (brown) at week 1 and 2 post-operation. Scale bar: 50 μm. (B) Quantitative analysis of TGF-β expression at week 1 and 2 post-operation. ^*^*P* < 0.05 and ^**^*P* < 0.01.
